# Liquid film condensation along a vertical surface in a thin porous medium with large anisotropic permeability

**DOI:** 10.1186/2193-1801-3-659

**Published:** 2014-11-06

**Authors:** Arthur S O Sanya, Christian Akowanou, Emile A Sanya, Gerard Degan

**Affiliations:** Laboratoire d’Energétique et de Mécanique Appliquées, LEMA EPAC, Université d’Abomey-Calavi, Cotonou, 01 B.P. 2009, Benin

**Keywords:** Liquid film condensation, Anisotropic in permeability, Thin porous medium, Brinkman-Darcy flow model

## Abstract

The problems of steady film condensation on a vertical surface embedded in a thin porous medium with anisotropic permeability filled with pure saturated vapour are studied analytically by using the Brinkman-Darcy flow model. The principal axes of anisotropic permeability are oriented in a direction that non-coincident with the gravity force. On the basis of the flow permeability tensor due to the anisotropic properties and the Brinkman-Darcy flow model adopted by considering negligible macroscopic and microscopic inertial terms, boundary-layer approximations in the porous liquid film momentum equation is solved analytically. Scale analysis is applied to predict the order-of-magnitudes involved in the boundary layer regime. The first novel contribution in the mathematics consists in the use of the anisotropic permeability tensor inside the expression of the mathematical formulation of the film condensation problem along a vertical surface embedded in a porous medium. The present analytical study reveals that the anisotropic permeability properties have a strong influence on the liquid film thickness, condensate mass flow rate and surface heat transfer rate. The comparison between thin and thick porous media is also presented.

## Introduction

Many investigations have been directed recently to attack film condensation in a porous medium in both steady (Cheng 1981; Chui et al. 1983; Nakayama and Koyama 1987; Vovos and Poulikakos 1987; Reeken et al. 1994) and transient problems (Cheng and Chui 1984; Ebinuma and Nakayama 1990; Masoud et al. 2000). Since the basic understanding of the transfer mechanisms of heat, momentum and mass in porous media during phase change have still developed, the two phase change flows have possible applications in geophysics and engineering problems such as geothermal energy, steam stimulation of oil fields, food drying and heat pipes problems (Ebinuma and Nakayama 1990). Al-Nimr and Alkam (1997) have studied steady film condensation on a vertical plate imbedded in a porous medium and have considered the Brinkman-Darcy model by neglecting the macroscopic and microscopic inertial terms to obtain analytical solutions for the three following requests: liquid film thickness, condensate mass flow rate and convective heat transfer coefficient. In the same way, Masoud et al. (2000) have adopted the Brinkman-extended Darcy model to found analytical solutions that describe the transient behavior of the three latter requests. The current results show the effect of the permeability of the porous material on several issues including the velocity profiles, the film thickness and the time required to reach steady state conditions.

Moreover, in all the above studies, the porous medium is assumed to be isotropic whereas, in the several applications, the porous materials are anisotropic (Degan et al. 1995). Despite this fact, film condensation in such anisotropic porous media has not received any attention.

Nevertheless, the study of natural convection in anisotropic porous medium is now available in the heat transfer literature (Kimura et al. 1993; Ni and Beckermann 1993; Zhang 1993; Degan and Vasseur 1996). Degan et al. (1995) have found analytical and numerical solutions for natural convection in a fluid-saturated porous medium filled in a rectangular cavity. In their work, the porous medium is assumed to be both hydrodynamically and thermally anisotropic. The principal directions of the permeability are oriented in a direction that is oblique to the gravity vector, while those of thermal conductivity coincide with the horizontal and vertical coordinate axes. The results showed that the analytical solutions can faithfully predict the flow structure and heat transfer for a wide range of the governing parameters.

In this paper, the problem of steady laminar film condensation on a vertical surface embedded in a thin porous medium with anisotropic permeability, with its principal axes oriented in a direction that is oblique to the gravity vector, based on the classical analysis by Nusselt (1916) is presented using the Brinkman-Darcy flow model (Tong and Subramanian 1985, Chan et al. 1970). Analogous to the work of Degan et al. (1995) who have studied the anisotropic behavior of the porous materials in the case of the natural convection in the neighborhood of vertical plate embedded in porous medium, the film condensation problem over a vertical plate embedded in a porous medium studied by Al-Nimr and Alkam (1997) is extended to cope with the reality which shows that the natural or industrial materials are anisotropic in their properties. With the consideration of the anisotropic permeability, the flow permeability tensor adopted is the same with previous report given by Degan et al (1995). The boundary-layer approximations are used only for the momentum equation in the porous liquid film region. The analysis aims to obtain closed-form expressions for the liquid film thickness, the condensate mass flow rate and the surface heat transfer rate.

### Mathematical formulation

**Figure 1:**
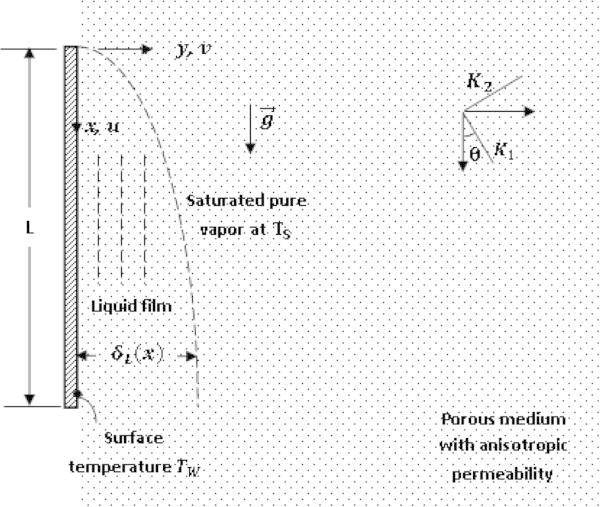
**Physical situation and coordinate system.**

The Figure 1 shows the development of a two dimensional laminar liquid film on a vertical plate of height *L* embedded in a porous medium saturated with a pure vapor. The *x* and *y* axes are aligned with the vertical and the horizontal coordinates, respectively. The anisotropy permeability of the porous medium is characterized by the anisotropy ratio K* = *K*_1_/*K*_2_ and the orientation angle *θ*, defined as the angle between the vertical direction and the principal axis with the permeability *K*_1_. It is assumed that the porous medium has large permeability. This assumption is valid if the thermal storage of the porous domain is neglected (Masoud et al. 2000). To investigate the problem of steady laminar film condensation about a vertical surface in a thin porous medium, the augmented latent heat of condensation and the vertical enthalpy inflow associated are two negligible and the following assumptions carried out by Al-Nimr and Alkam (1997) after Nusselt (1916) will be made:

-  Constant properties are assumed for the liquid film;
-  The liquid and solid porous domains are in local equilibrium;
-  The temperature distribution is in steady state and lower than the saturation temperature of the liquid film;
-  The gas is assumed to be a pure vapor at a uniform temperature equal to *T*_*S*_ and no conduction of heat is assumed to take place at the liquid-vapor interface.
-  The local film thickness is much larger than the pore or particle size. This makes it possible to use the local volume-averaged treatment of the current heterogeneous fluid-solid system (Kaviany 1995).
-  The problem is gravity dominated and capillarity effects are then neglected. As a result, the two-phase region, which contains a solid matrix saturated with vapor and liquid, is absent.
-  The shear stress at the liquid-vapor interface is assumed to be negligible ; there is no need to consider the vapor velocity or thermal boundary layers.
-  The heat transfer across the film occurs only by conduction, in which case the liquid’s temperature distribution is assumed to be linear.
-  The boundary-layer approximation is valid in the momentum equation for a porous liquid film. However, both macroscopic and microscopic inertial terms are assumed to be negligible (Kaviany 1995).

Under the aforementioned assumptions, the governing equation for the porous liquid film is given as (Tong and Subramanian 1985):
1

Where *P* is the pressure in the porous medium, *ρ*_*L*_ the density of the liquid film,  the gravitational acceleration vector, *μ*_*L*_ the dynamic viscosity of the liquid film, *μ*_*L*,*e*_ the dynamic viscosity of the liquid film in the porous medium,  the velocity vector of the liquid film in the porous medium and  the inverse symmetrical second-order permeability tensor defined in the cartesian frame of reference as:
23a3b3c3d

The final expression for the equation (1) is a single momentum (4) obtained by eliminating the pressure term by making use of equation (5):
45

Equation (7) has the boundary conditions:
6a

and
6b

### Dimensional analysis

In the boundary layer regime, the liquid film motion is restricted to a thin layer *δ*_*L*_ along the vertical plate. The condition of existence of the vertical boundary layer hypothesis is formulated when *δ*_*L*_ ≪ *L* and is valid when the motion of the liquid along the vertical surface is considered to be laminar in the x direction. So it is clear, from the momentum equation (4), that one may use the boundary-layer hypothesis only when the following conditions are satisfied:
789101112

These conditions (7-12) arise because of the using of the vertical boundary layer hypothesis which impose that the equation (4) much be only dependent from the motion in x direction, that is to say the terms in y direction must be negligible with respect to the terms in the x direction to have the thin boundary layer along the vertical surface. Some examples are given in Bejan (1984).

Then, under the boundary-layer approximations (7) to (12), the governing momentum equation (13) for the porous liquid film may be read:
13

Following Bejan (1984) and recognizing *L* and *δ*_*L*_ as the *x* and *y* scales, respectively, in the boundary layer of interest (*δ*_*L*_ ≪ *L*), the conservation equation (13) requires the following balances:
14

From the conservation of mass for the porous liquid film, we have:
15

Solving the balances between equations (13) and (15) for *δ*_*L*_, *u*_*L*_ and *v*_*L*_, we obtain the following results:
161718

Where the following non-dimensional terms of the porous medium are in used:

-  
19
-  
20
-  
21

The stream function *ψ*_*L*_ related to the velocity components is defined such that the continuity equation is automatically satisfied by:
22a

and
22b

The scale for the stream function can be obtained as follows:
23

Taking into account the previous scales obtained in this case, the condition of existence of the vertical boundary layer hypothesis formulated by *δ*_*L*_ ≪ *L* becomes *ϵ Da*^- 1^ ≫ *a*. Moreover, making use of equations (7) to (12), the boundary-layer hypothesis is valid only when the conditions (24) and (25) are satisfied:
2425

### Resolution

The resolution of equation (13) can permit to determine the velocity profile, the condensate mass flow rate, the liquid film thickness and the convection heat transfer coefficient.

So, making transformation of equation (13) to equation (26) which solution is obtained in analogous to Al-Nimr and Alkam (1997), we found the expression (28) for the velocity component *u*_*L*_ on the *x* axis:
26

Where *A* and *C* are the constants such that:
27a

and
27b

The above equation (26) with boundary conditions (6) can be integrated to give:
28

When K* = 1, i.e., *a* = 1, equation (28) becomes identical to solution obtained by Al-Nimr and Alkam (1997) in the case of condensation with negligible microscopic inertial term.

The condensate mass flow rate per unit width Γ(*x*) can be obtained in terms of an integral involving the velocity profile as:
29

Where *B* is the liquid film width.

Substituting the velocity distribution given in equation (28) into equation (29), the resulting expression (30a) can be rearranged to obtain finally the dimensionless condensate mass flow rate per unit width in the form (30b):
30a30b

For the thin porous domain, where *K*_1_ → ∞ and *A* → 0 and the notation that for small *A*, we have as the same expressions doing by Masoud et al. (2000):
31a

and
31b

Substituting equations (31a,b) into equation (30b) that implies finally for the dimensionless condensate mass flow rate per unit width the following expression:
32

Where the dimensionless parameter *σ* is defined as:
33

The energy balance which states that the rate of energy release due to condensation at the vapor-liquid interface must equal the rate of heat transfer from the film to the wall can be considered to determine the film thickness *δ*_*L*_(*x*).

To this end, we write equation (34) and substituting the expression (29) into this to find the relation (35):
3435

Now, inserting equation (30a) into equation (35) yields:
36

Integrating equation (36), for a specified surface temperature *T*_*W*_, from *x* = 0, where *δ*_*L*_ = 0, to any axial location *x* yields:
37

For small *A*, equation (37) becomes:
38

This equation (38) assumes six different roots from which the only acceptable one is the positive real root given by the same expression as Al-Nimr and Alkam (1997), namely:
39

Where *R* and *Q* are the parameters such that:
40a40b

We now multiply the expression (39) by the factor  in order to obtain the non-dimensional expression for the liquid film thickness. We have finally found that this dimensionless equation is:
41

Where the following dimensionless parameters of the porous liquid film are defined as:

-  
42a
-  
42b

It is important to note that the parameter *σ* in equation (41) should satisfy the following condition:
42c

Then we can write the expression (42d) for *σ* and the positive parameter *ω* must satisfy the condition (42e):
42d42e

Substituting equation (42d) into equation (41) yields:
43

The local convection heat transfer coefficient is given as the same expression adopted by Al-Nimr and Alkam (1997):
44

The surface heat flux from the edge of the liquid film to the vertical surface embedded in the porous medium is easily obtained by utilizing equation (44) and represented in terms of the local Nusselt number:
45

Substituting equations (41) and (44) into (45), one obtains finally:
46

## Results and discussion

In what follows, the main results of our study related to the effects of anisotropic permeability parameters based on the Brinkman-Darcy flow model are compared essentially with Cheng’s steady solution (Cheng 1981) based on the Darcy flow model and Ebinuma’s steady solution (Ebinuma and Nakayama 1990) based on the Ergun model (Ergun 1952) and Sanya’s steady solution (Sanya et al. 2014) for thick porous medium with anisotropic permeability based also on the Brinkman-Darcy flow model.

**Figure 2:**
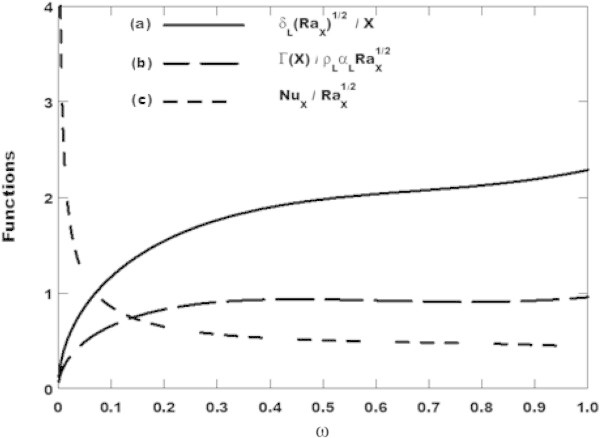
**Variation of (a) film thickness, (b) condensate mass flow rate per unit width and (c) Nusselt number with the parameter** **ω** **for** ***Ja*** **= 2.85,** ***θ*** **= 30** ***°*** **and** ***K*** * = 2.5.

The following values for the parameter *ω* from zero to one in Figure 2 may be easily extracted from the equation (42c) which can be written in the form of the expression (42d) to find the condition (42e). As conclusion, the latter condition should be satisfied to have the Brinkman-Darcy flow model for the thin porous medium with anisotropic permeability.

In Figure 2, it is observed that, when the anisotropic parameters are held constant, for example, for *θ* = 30*°* and K* = 2.5, the film thickness and the condensate mass flow rate per unit width are both increased when the parameter ω is increased while the Nusselt number is decreased.

**Figure 3:**
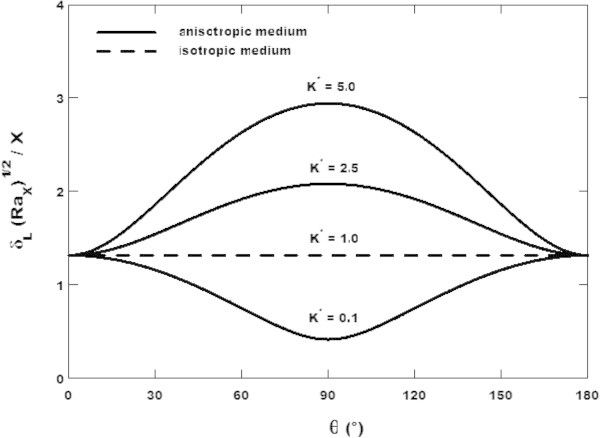
**Effect of anisotropy orientation angle** ***θ*** **on the film thickness for** ***Ja*** **= 2.85 and** ***ω*** **= 0.2 and various values of permeability ratio.**

Equation (43) is plotted in Figure 3 where it is shown that the dimensionless thickness of the liquid film increases as anisotropy orientation angle *θ* is increased from zero towards 90° for K* > 1 and decreased for K* < 1 while the reverse effect is observed from 90° towards 180°. The same result is found by Sanya et al. (2014) in their work on film condensation on a vertical surface embedded in a thick porous medium with anisotropic permeability. Thus, one can limit the discussion to 0 ≤ *θ* ≤ 90*°*. When K* > 1, the dimensionless thickness of the liquid film is maximum at *θ* = 90*°* and minimum at *θ* = 0 while the opposite result is observed for K* < 1. It follows from these results that the orientation of the principal axis with higher anisotropic permeability of the thin porous medium perpendicular to the gravity vector implies a maximum liquid film thickness on the vertical surface. When K* = 1, the film thickness remains constant with the increasing of anisotropy orientation angle *θ* which corresponds to the properties of isotropic porous medium.

**Figure 4:**
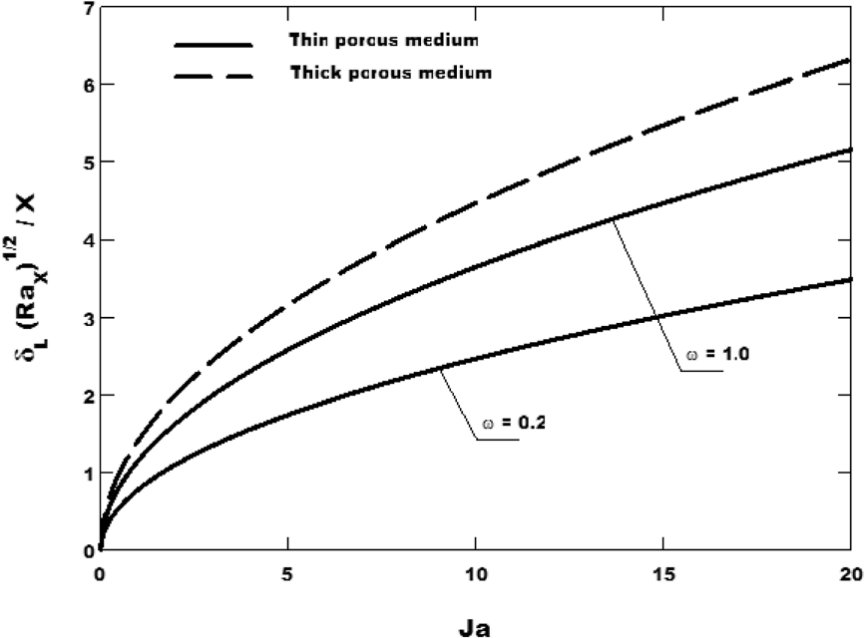
**Variation of the film thickness with the Jacob number for** ***θ*** **= 30** **°** **and** ***K*** * = 1.0 **and two values of the parameter** ***ω*** **.** Comparison between the thin and the thick porous media.

The variation of the dimensionless thickness of the liquid film with the Jacob number is plotted in Figure 4 where it is shown that the liquid film increases as *Ja* is increased. This result is similar to that obtained analytically by Cheng (1981) using the Darcy flow model and Ebinuma and Nakayama (1990) using the Ergun model (Ergun 1952). Moreover, it is seen that the porous medium has the effect of increasing the condensate’s film thickness as similar to the result obtaining by Al-Nimr and Alkam (1997). Then, it is clear from this figure that large anisotropic permeability of the thin porous medium has the effect of decreasing the liquid film thickness as compare to the thick porous medium studied by Sanya et al. (2014).

**Figure 5:**
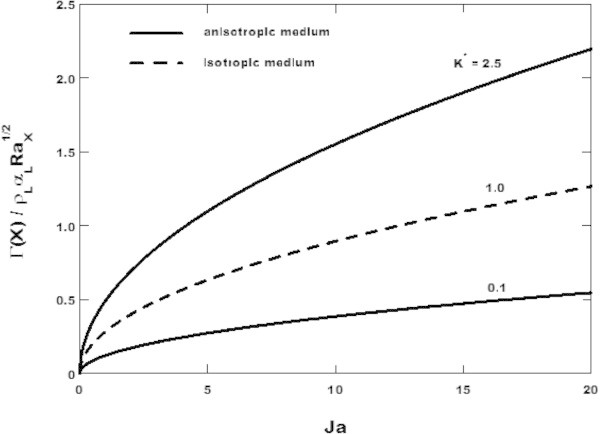
**Variation of the condensate flow rate per unit width with the Jacob number for** ***θ*** **= 30** ***°*** **and** ***ω*** **= 0.2 and various values of the permeability ratio.**

Figure 5 shows the growth of the condensate flow rate per unit width with respect to the Jacob number for the permeability ratio K* = 0.1, 1.0 and 2.5 respectively. The isotropic properties of the porous medium are obtained when K* = 1.0 which is plotted in dashed line in Figure 5. As is clear from this figure, the anisotropic in permeability has the effect of increasing the condensate flow rate per unit width for K* > 1.0 and the effect of decreasing the latter for K* < 1.0. This behavior comes from the fact that, according to Eq. (32), when the parameters *ω*, *θ* and *Ja* are held constant, the condensate flow rate per unit width depends solely on the dimensionless liquid film thickness and is proportional to this latter which is increased when K* > 1.0 and decreased when K* < 1.0 as is showed in Figure 3.

**Figure 6:**
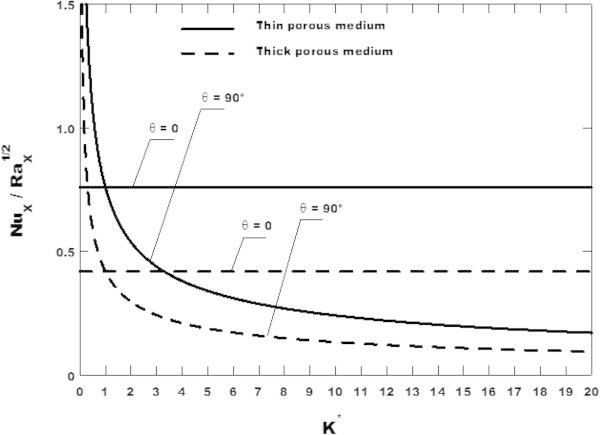
**Effect of the anisotropic permeability ratio on Nusselt number for** ***Ja*** **= 2.85** **and** ***ω*** **= 0.2** **and various values of anisotropy orientation angle** ***θ*** **.** Comparison between the thin and the thick porous media.

Figure 6 shows the effect of the anisotropic permeability ratio on Nusselt number given by Eq. (46) for fixed values *Ja* = 2.85 and *ω* = 0.2 and various values of anisotropy orientation angle *θ*. In this Figure, for K* > 1.0, when the anisotropy orientation angle *θ* varies from zero to 90*°*, the Nusselt number decreases for both the thin and the thick porous media and the reverse effect is observed for K* < 1.0. However, this decreasing is more important for the case of the thick porous medium. Another important observation in Figure 6 can be made for isotropic properties for which it is easily seen from equation (3a) that *a* = 1 for *θ* = 0. Thus, when *θ* = 0, the Nusselt number is found to be independent of K*. This result is qualitatively similar to that obtained analytically and numerically by Degan et al. (1995), who has studied the effect of anisotropic permeability on the convective heat transfer in a fluid saturated porous medium filled in a rectangular cavity heated with anisotropic thermally. The principal directions of the permeability are oriented in a direction that is oblique to the gravity vector, while those of thermal conductivity coincide with the horizontal and vertical coordinate axes.

**Figure 7:**
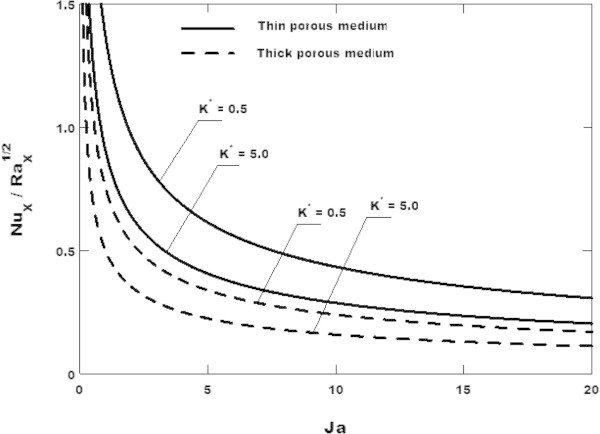
**Variation of the Nusselt number with the Jacob number for** ***θ*** **= 30** **°** **and** ***ω*** **= 0.2** **and various values of the anisotropic permeability ratio.** Comparison between the thin and the thick porous media.

The variation of the Nusselt number is plotted in Figure 7 where it is observed that the Nusselt number decreases with the anisotropic permeability ratio for both the thin and the thick porous media. This implies that porous medium has the effect of decreasing the surface heat transfer rate and as a consequence the liquid film thickness increases. This can be explain by the following two reasons carried out by Masoud et al. (2000): (1) The enhancement of conduction heat transfer in the transverse direction of the liquid film thickness, especially by using solid matrix having high thermal conductivity and (2) the reduction in the void volume, due to the reduction in the permeability, leaves less void space per unit total volume for the condensate liquid, and as a result, the condensate liquid film becomes much thicker to contain the same amount of the condensate liquid.

## Conclusion

In this paper, the analyses by Al-Nimr and Alkam (1997) have been extended to take account the anisotropic properties of the porous medium, using the flow permeability tensor given by Degan et al. (1995). The Brinkman-Darcy flow model has been developed to investigate the steady film condensation on a vertical surface embedded in a thin porous medium with anisotropic permeability whose principal axes are non-coincident with the gravity vector. Within the boundary layer approximations, dimensionless-form expressions have been obtained that describe the steady behavior of the liquid film thickness, condensate mass flow rate and surface heat transfer rate. The relative significance of the anisotropic properties may be accounted, in general manner, by introducing the two anisotropic parameters named as the permeability ratio K* and the orientation angle *θ* of the principal axes. Both the permeability ratio and orientation angle of the principal axes have a strong influence on the liquid film condensation in the anisotropic porous media. As is can be noticed in this study, isotropic properties of the porous medium come with *a* = 1, that is for *θ* = 0 (or K* = 1.0), and the dimensionless thickness of the liquid film and the Nusselt number are found to be independent of K* (or *θ*). As the two anisotropic parameters increase, when *θ* varies from zero towards 90*°*, the surface heat transfer rate decreases while the liquid film thickness and the condensate mass flow rate grow, for both the thin and the thick porous media and this behavior is more drastic in the case of the thick porous medium.

### Nomenclature

*a*, *b*, *c*, Constants, equations (3a,b,c)

*B*, Liquid film width (*m*)

*C*_*P*_, Specific heat capacity of fluid at constant pressure (*J. kg*^- 1^. *K*^- 1^)

*Da*, Darcy number, equation (19)

*g*, Gravitational acceleration (*m. s*^- 2^)

*h*_*Lv*_, Latent heat of condensation (*J. kg*^- 1^)

*h*_*x*_, Local convective heat transfer coefficient (*W. m*^- 2^*K*^- 1^)

*Ja*, Jacob number, equation (42a)

, Flow permeability tensor, equation (2)

*K*_1_, *K*_2_, Flow permeability along the principal *x* and *y* axes, respectively (*m*^2^)

K*, Anisotropic permeability ratio, equation (3d)

*k*, Thermal conductivity (*W. m*^- 1^*K*^- 1^)

*L*, Height of the surface (*m*)

*Nu*_*x*_, Local Nusselt number, equation (45)

*Ra*_*L*_, Rayleigh number, equation (20)

*Ra*_*x*_, Local Rayleigh number, equation (42b)

*T*, Temperature (*K*)

V, Velocity of the liquid film in the porous medium (*m. s*^- 1^)

*u*, *v*, Velocity components in *x*, *y* directions (*m. s*^- 1^)

*P*, Pressure (*Pa*)

*x*, *y*, Cartesian coordinates (*m*)

Greek symbols

*α*, Thermal diffusivity (*m*^2^. *s*^- 1^)

*δ*_*L*_, Liquid film thickness (*m*)

*ϵ*, Porosity of the porous medium

*μ*, Dynamic viscosity (*kg. m*^- 1^. *s*^- 1^)

Γ, Condensation mass flow rate per unit length (*kg. s*^- 1^. *m*^- 1^)

ω, Positive parameter defined in equation (42e)

*ψ*, Stream function (*m*^2^. *s*^- 1^)

*ρ*, Density of the fluid (*kg. m*^- 3^)

*σ*, Dimensional parameter defined in equation (42d)

θ, Orientation angle of the principal axes (*°*)

Superscripts

*, Dimensional quantities

Subscripts

*e*, Effective properties due to the presence of the porous domain.

*L*, Liquid region

*s*, Saturated properties

*v*, Vapor region

*w*, Refers to the vertical surface.
